# Corticospinal Plasticity in Bilateral Primary Motor Cortices Induced by Paired Associative Stimulation to the Dominant Hemisphere Does Not Differ between Young and Older Adults

**DOI:** 10.1155/2017/8319049

**Published:** 2017-09-24

**Authors:** Daina S. E. Dickins, Marc R. Kamke, Martin V. Sale

**Affiliations:** ^1^Queensland Brain Institute, University of Queensland, Brisbane, QLD, Australia; ^2^Division of Physiotherapy, School of Health and Rehabilitation Sciences, University of Queensland, Brisbane, QLD, Australia

## Abstract

Older adults have been shown to exhibit a reduction in the lateralization of neural activity. Although neuroplasticity induced by noninvasive brain stimulation has been reported to be attenuated in the targeted motor cortex of older adults, it remains possible that the plasticity effects may instead manifest in a more distributed (bilateral) network. Furthermore, attention, which modulates neuroplasticity in young adults, may influence these effects. To address these questions, plasticity was induced in young (19–32 years) and older (65–78 years) adults using transcranial magnetic stimulation (TMS) paired with peripheral nerve stimulation. The plasticity effects induced by this paired associative stimulation (PAS) protocol in the targeted and nontargeted hemispheres were probed using TMS-induced motor-evoked potentials (MEPs) recorded from the abductor pollicis brevis (APB) muscle of each hand. PAS-induced effects were highly variable across individuals, with only half of the participants in each group demonstrating the expected increase in MEP amplitude. Contrary to predictions, however, PAS-induced corticospinal plasticity manifests predominately in the targeted hemisphere for both young and older adults. Attention to the target hand did not enhance corticospinal plasticity. The results suggest that plasticity does not manifest differently across bilateral corticospinal pathways between young and older adults.

## 1. Introduction

Advancing age is associated with neurobiological change throughout the brain that can impact neuronal structure and function (see [1, 2] for review). An interesting example of this is the finding that older compared to younger adults demonstrate proportionately more neural activity in homologous regions of the hemisphere ipsilateral to the moving hand during the performance of a motor task [3, 4]. Given that older adults also demonstrate impaired learning and memory, this finding raises the question, is there a relationship between changes in the laterality of neural activity (i.e., distribution of neural activity across hemispheres) and impaired learning and memory? A possible consequence of ageing is that the population of cells responsible for motor learning (henceforth referred to as a cell assembly [5]) might spread across an increasingly bilateral neural network in older adults [3, 4]. As learning and memory are underpinned by neuroplasticity, research has begun to answer this question by examining the manifestation of neuroplasticity across the hemispheres in young and older adults. However, few studies have directly examined whether the manifestation of plasticity affecting bilateral motor cortices is altered in older adult humans.

Neuroplasticity is defined as the ability of the brain to undergo enduring morphological or functional change in response to the demands of its environment [6]. An important mechanism of neuroplasticity is synaptic plasticity [7], which plays an important role in learning and memory by governing the number and strength of connections within a cell assembly [5]. Little is known about how age-related change impacts synaptic plasticity in older adult humans. One approach to indirectly probe synaptic plasticity in humans is by applying noninvasive brain stimulation, such as transcranial magnetic stimulation (TMS). When applied over the hand region of the primary motor cortex, TMS can activate underlying neural populations and, via the corticospinal pathway, ultimately result in a motor response in a contralateral hand muscle known as a motor-evoked potential (MEP). The peak-to-peak amplitude of the MEP can be quantified using electromyography (EMG). Critically, one mechanism by which TMS impacts motor output cells is by transsynaptic activation, and therefore changes in synaptic efficacy of the target neurons can lead to changes in MEP amplitude [8–11]. Few studies have investigated how plasticity induction applied to the motor cortex of one hemisphere impacts on the excitability of motor cortical neurons in the contralateral, nonstimulated hemisphere (henceforth referred to as the “manifestation” of plasticity) in young and older adults [12, 13]. The results of these studies have been equivocal. For example, following training on unimanual simple and complex motor tasks, corticospinal excitability of bilateral primary motor cortical regions has been found to increase bilaterally in both young and older adults [12]. However, following the application of brain stimulation to the target hemisphere, corticospinal excitability did not increase significantly in either the targeted or nontargeted hemisphere in young or older adults [13].

Paired associative stimulation (PAS [8]) is an intervention that has been shown to induce changes in the human motor cortex with characteristics comparable to associative long-term potentiation (LTP) plasticity in animal models. Studies in animal models have demonstrated that synaptic plasticity can be dependent on the synchrony and temporal order of activity of neighbouring neurons [14]. The efficacy of the communication between neighbouring neurons is increased (potentiated) with synchronous activity or decreased (depressed) with asynchronous activity. Consistent with this observation, synchronous and repetitive pairings of low-frequency single-pulse TMS with peripheral nerve stimulation (i.e., the PAS protocol), which lead to coincident inputs to motor cortex, have also been shown to induce LTP-like increases in corticospinal excitability in humans [8, 9]. Long-term depression- (LTD-) like decreases in corticospinal excitability have also been demonstrated when PAS pairings are asynchronous and do not lead to temporally coincident inputs to the primary motor cortex (M1) [9]. To date, no studies that have investigated age-related changes in the manifestation of plasticity across the motor cortices have implemented PAS [8]. Importantly, PAS may induce plasticity that is more similar to motor training than the noninvasive brain stimulation technique used in our previous study [13] due to the involvement of sensory input from the peripheral nerve stimulation.

Notably, the manifestation of plasticity might also be influenced by cognitive processes taking place during the plasticity intervention. Moreover, this interaction may differ between young and older adults. Previous research indicates that TMS-induced effects in the motor cortices of young adults can be impacted by concurrently undertaking a cognitive task [15–18]. For example, allocating spatial attention towards the hand targeted by the TMS intervention results in increased excitability in the corticospinal pathway innervating the target muscle relative to conditions in which attention was directed elsewhere [15–19]. These effects may differ in older adults, who are more susceptible to a neural change in the frontal and parietal regions [20], which are known to play a critical role in controlling attentional processes [21]. No studies to date have directly investigated the influence of attention in young and older adults on the manifestation of corticospinal plasticity across bilateral motor cortices. Therefore, the current study also tested the hypothesis that attention to the target hand enhances PAS-induced corticospinal plasticity in the target pathway of both young and older adults relative to when attention is allocated to the nontargeted hand.

The aim of this research is to determine whether the manifestation of PAS-induced plasticity across the hemispheres differs between young and older adults. It is hypothesised that PAS-induced plasticity will manifest over an increasingly bilateral network in older compared to younger adults, which will be reflected by similar changes in MEPs post-PAS in both the target and nontargeted motor cortices. An additional question is the extent to which the allocation of spatial attention to either the target or nontarget hand influences the manifestation of plasticity in young and older adults. Given the age-related change in attentional networks that appear to result in attentional deficits, it is expected that the influence of attention will differ between young and older adults such that attention will facilitate LTP-like plasticity in the target hemisphere when attention is allocated to the target but not the nontarget hand for young but not older adults. To index the degree of age-related change in the cognitive and motor systems of participants and to examine associations between these characteristics and the manifestation of corticospinal plasticity in the two age groups, measures of cognitive and motor ability, physical activity, and psychological factors will also be included. It is expected that older adults will have lower levels of performance compared to the young adults on the cognitive and motor tasks.

## 2. Methods

### 2.1. Participants

A total of 40 participants were involved in the study; 20 between the ages of 19 and 32 years (young group; *M* = 24.40, SD = 3.86, males = 10) and 20 between the ages of 65 and 78 years (elderly group; *M* = 69.55, SD = 3.99, males = 10) were tested. All participants were right-handed as determined by The Edinburgh Handedness Inventory (young *M* = 81.34, SD = 18.08, older *M* = 84.54, SD = 19.12; [22]). Prior to commencement of testing, all participants completed a TMS safety-screening questionnaire [23, 24] and provided fully informed written consent. All procedures were approved by The University of Queensland's Medical Research Ethics Committee. Individuals with neurological disease or damage, epilepsy, history of head injury or psychiatric disorder, or who were taking neuroactive medications were excluded from the study. All participants had normal or corrected to normal visual acuity. There were no adverse reactions to TMS.

### 2.2. Cognitive and Psychological Assessment

Participants were administered with a battery of cognitive tests which measured attention, perceptual speed, cognitive flexibility, executive control, response inhibition, working memory, and executive capacity. These tests included the Stroop test [25], and two components of the fourth edition Wechsler Adult Intelligence Scale (WAIS-IV; [26]), the digit span test, and the Logical Memory Test. In addition, each participant's psychological well-being and connectedness to social groups were measured using the Satisfaction with life scale [27] and the Multiple Identities Scale [28]. The General Physical Activity Questionnaire (GPAQ; [29]) was used to measure the average physical activity undertaken by each participant in a typical week. Participants also completed a demographic questionnaire denoting the occupation in which they spent most time and the highest level of formal education achieved. Educational attainment was recorded on an ordinal scale from 0, representing “no schooling completed,” to 11, denoting a “doctorate degree.”

### 2.3. Motor Assessment

Participants were required to perform six different timed tapping tasks, performed either with one hand (unilateral) or with two hands (bilateral). All tapping tasks were completed on a standard computer keyboard and were repeated three times in separate blocks. Participants completed five practice trials prior to the first test of each task in block one to ensure they had learnt the required motor actions and sequences. The six tasks were the following: *right index—*tap the “o” key with the right index finger (task 1); *left index—*tap the “w” key with the left index finger (task 2); *right alternate—*tap the “o” and “p” keys alternately using the index and middle fingers of the right hand (task 3); *left alternate—*tap the “w” and “q” keys alternately using the index and middle fingers of the left hand (task 4); *index alternate—*tap the “o” and “w” keys alternately using the index fingers of the right and left hands (task 5); *bilateral alternate—*tap the “o,” “q,” “w,” and “p” keys in that sequence using the right index finger, left middle finger, left index finger, and right middle finger, respectively (task 6). Performance was quantified based on the number of accurate sequences completed in 15 seconds [30].

### 2.4. Transcranial Magnetic Stimulation (TMS)

TMS was administered using a figure-of-eight-shaped coil with a wing diameter of 70 mm (#9925-00), connected to a Magstim 200^2^ stimulator (Magstim Co., UK). The coil was placed tangentially on the scalp with the handle pointing towards the back of the head, angled 45 degrees from the midline, and was moved systematically in a grid-like pattern until the motor hotspot was located. The motor hotspot was defined as the optimal position on the scalp for evoking the largest and most consistent MEP (peak-to-peak amplitude) in the target muscle, the *abductor pollicis brevis* (APB) muscle of the left and right hands. Stimulation occurred approximately every 5 seconds at an intensity sufficient to evoke a clear MEP in the target muscle. A frameless infrared stereotaxic neuronavigation system (Visor 1, ANT, Netherlands) was used to record the location and angle of the coil for each hotspot, enabling these to be reproduced within an experimental session.

Following determination of the hotspot, resting motor threshold (rMT) was obtained for the cortical representation controlling the left and right APB. The rMT was defined as the minimum TMS intensity (reported as a percentage of maximum stimulator output, % MSO) that evoked a MEP of at least 50 *μ*V in at least 3 out of 5 consecutive trials. The intensity of the TMS was adjusted using a staircase (two-down, one-up) procedure until the criterion was met. Following this, TMS test intensities were established for the left and right APB. The test intensity was defined as that required to evoke an average MEP of approximately 1 mV (peak-to-peak) in the resting muscles. On average, this intensity equated to 121% of rMT for the right (target) APB and 123% of rMT for the left (nontarget) APB (see Table 1 for details). Twenty-one pulses were administered at the test intensity every 5 ± 1 seconds at each time point. All trials, except the first pulse and trials displaying excessive muscle activity, were utilized to quantify average MEP amplitude at baseline (i.e., pre-PAS) and at the four timepoint post-PAS (at 5, 15, 25, and 35 minutes post).

### 2.5. Electromyography (EMG)

Activity from the targeted and nontargeted APB was recorded using surface EMG. Disposable 24 mm silver-silver chloride electrodes were used, with the active electrode placed on the belly of the APB muscle of the left and right hands and reference electrodes on the metacarpophalangeal joint of the respective thumb. MEP data were amplified (×1000), filtered (20–2000 Hz), and sampled at 2000 Hz using a NeuroLog System (Digitimer, UK), National Instruments Data Acquisition Interface (BNC-2110, National Instruments, USA), and custom Matlab software (Mathworks, USA). Individual sweeps were sampled from 500 ms before stimulation to 500 ms after stimulation and stored for off-line analysis. Muscle activity was visually monitored throughout the experiment using a digital oscilloscope. If activity occurred during a trial, participants were verbally prompted to relax, and any trials containing muscle activity were discarded from subsequent analyses.

### 2.6. Paired Associative Stimulation (PAS)

Plasticity was induced in the targeted cortical region with PAS, which has been shown previously to induce reliable increases in cortical excitability in young adults [31]. One hundred and thirty-two pulses of TMS were administered to the left hemisphere (“target M1”) at the predetermined test intensity, spaced 4 to 6 seconds apart. Each pulse was paired with peripheral nerve stimulation, which occurred 25 ms prior to each TMS pulse. This interval was chosen as it has been shown to induce LTP-like increases in corticospinal excitability [9]. Peripheral nerve stimulation was applied to the median nerve at the wrist using a constant current stimulator (Digitimer DS7A) and bar electrode (200 *μ*s pulse width; cathode proximal). The intensity of the peripheral nerve stimulation was adjusted to produce a small (~200 *μ*V) but clearly discernable motor response in the right APB.

### 2.7. Visual-Spatial Attention Manipulation

Sustained spatial attention was manipulated across the two PAS sessions. Participants were instructed to overtly attend to a light emitting diode (LED) placed on the thumb, just above the interphalangeal joint. Attention was directed to the targeted and nontargeted thumb in separate sessions; the order of which was counterbalanced across individuals. Participants were tasked with making a verbal response each time they detected two brief, consecutive interruptions (“OFF” periods) to the continuously lit LED. Targets appeared on average every 10 seconds, but were jittered randomly, such that the interval between targets varied between 5 and 30 seconds. Targets were timed such that participants did not respond during a PAS pulse. Targets were also intermingled with nontargets (single “OFF periods”) that were also jittered randomly. The temporal presentation of targets remained constant across the attend target and attend nontarget conditions. The attention task was performed for the duration of the PAS intervention (11 mins).

### 2.8. Experiment Design and Procedure

**Figure 1:**
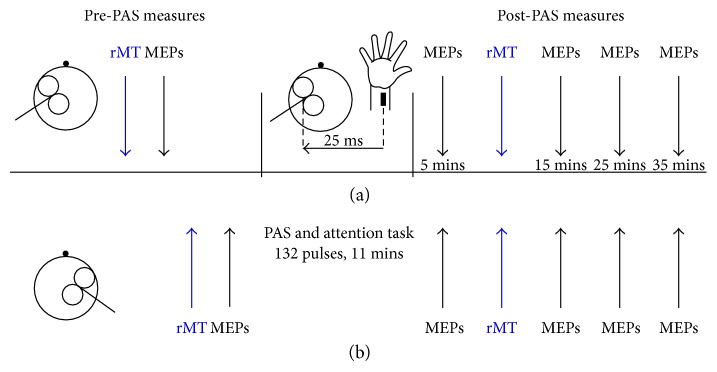
Time course of experiment. Row A denotes the time course for the left hemisphere (target M1), and row B denotes the time course for the right (non-target) hemisphere. MEPs were acquired by stimulating the left M1 followed by the right M1 pre-PAS and 5, 15, 25, and 35 minutes post-PAS. Resting motor threshold (rMT) was determined at two time points for each hemisphere, once before and once following PAS.

Participants completed two PAS sessions that took place at least 24 hours apart at similar times of day to minimise any circadian-related effects on plasticity [31]. Cognitive and motor assessments took place in an additional session. During the PAS sessions, participants were seated comfortably with their forearms resting in a pronated position on a cushioned desk. The skin of both hands was cleaned thoroughly to minimise skin impedance, and electrodes were placed in position. An eye tracker was used throughout the pre-PAS and post-PAS measures to ensure that participants' eyes remained open. The timeline of the PAS sessions is depicted in Figure 1. Single-pulse TMS was applied to the target and nontarget M1s to locate the motor hotspot and to quantify corticospinal excitability before PAS. This was followed by a brief practice of the attention task, which was then undertaken during PAS. Following PAS, single-pulse TMS was administered at the test intensity, first to the target and then to the nontarget hemisphere, to obtain post-PAS MEPs at 5, 15, 25, and 35 minutes. rMTs were remeasured between the 5 and 15 min post-PAS MEP measures.

### 2.9. Data Processing and Analyses

To identify age differences in cognition, psychological well-being, and physical activity, scores on these measures were subjected to an independent samples *t*-test. Motor performance was assessed across age groups and tasks with a 2 × 6 mixed analysis of variance (ANOVA) with factors of age (young and older) and task (right index, left index, index alternate, right alternate, left alternate, and bilateral alternate). Two young adults did not complete the session in which the motor performance, psychological, and cognitive data were collected. Performance on the spatial attention task, undertaken during PAS, was quantified by comparing the total number of errors made during the task across groups and conditions. Total error was calculated by summating the number of targets missed (misses) with the number of false positives for each participant. Performance was compared between age groups and attention conditions using a 2 × 2 mixed ANOVA testing the factors attention (attend right and attend left) and age (young and older).

Corticospinal plasticity was assessed using EMG data that were analysed offline using custom Matlab software. The first pulse from each block of 21 MEPs was removed, as were trials containing muscle activity clearly above background noise (cut off estimate ~30–40 *μ*V) in the 100 ms prior to TMS. Prestimulus activity levels were assessed visually. The remaining trials in each block were averaged for each participant. Baseline MEPs and test intensities were subjected to a 2 × 2 × 2 mixed ANOVA with the factors of hemisphere (target and nontarget), attention (attend right and attend left), and age (young and older). Pre- and post-PAS rMTs were subjected to a 2 × 2 × 2 × 2 ANOVA with the between subject factor of age (young and older) and the within subject factors of hemisphere (target and nontarget), time (pre and post), and attention (attend right and attend left). Post-PAS MEP amplitudes were expressed as the average percentage change from each participant's pre-PAS baseline. Post-PAS MEP change in the APB muscles was compared using a 2 × 2 × 2 × 4 mixed ANOVA with factors of age (young and old), hemisphere (target and nontarget), attention (right and left), and time (5, 15, 25, and 35 mins post-PAS). One young participant failed to complete the 35 minute post-PAS measure in the attend right condition. This missing data point was replaced with the overall average of the 5-, 15-, and 25-minute post-PAS measures in the attend right condition for that individual combined with the average of all the young adults at 35 mins post-PAS. SPSS software was used to conduct the analyses. A priori significance was set at *p* < 0.05. Mauchly's test of sphericity was used to determine violations of sphericity, and the Greenhouse-Geisser correction was applied where the sphericity assumption was violated. Cohen's *d* and Partial eta-squared were calculated and were reported to indicate effect sizes.

Because initial analyses revealed that no PAS-induced effects were evident at the group level, and individuals showed large variability in their responses to PAS, participants were classified into two groups based on the direction of MEP change induced separately in the attend right and attend left conditions. This was done to examine the manifestation of plasticity responses across the hemispheres in young and older adults. Individuals with an increase in MEP amplitude (>10% increase) averaged over all post-PAS time points in the target muscle were classified as “LTP-like responders” [32]. Individuals with a decrease in MEP amplitude (>10% decrease) in the target muscle post-PAS were classified as “LTD-like responders.” Responder classification was included as an additional factor in an exploratory 2 × 2 × 4 ANOVA for the attend right and attend left conditions separately. The results of the analyses for LTP-like and LTD-like responders were unaffected by the criterion used (i.e., above or below 0%, 10%, or 20%; see [33]). All main effects and interactions were followed up with paired comparisons using Bonferroni corrections and two-tailed *t*-tests.

## 3. Results

### 3.1. Age Differences in Cognitive, Psychological, and Physical Activity Assessments

Table 2 displays the means, standard error of the means, and *t*-test results for the cognitive, physical, and psychological results. Significant effects are shown with an asterisk. Older adults reported significantly less sedentary activity and more physical activity per week than young adults (Table 2). Young adults demonstrated greater accuracy in the logical memory immediate and delayed recall task than older adults. In addition, in comparison to young adults, older adults experienced significantly greater cognitive interference between the two components of the Stroop task. The highest level of educational attainment was similar for both young and older adults, with the most common level of education being a bachelor's degree. There were no other significant differences in the performance of young and older adults across the different cognitive and psychological measures.

### 3.2. Age Differences in Motor Performance

**Figure 2:**
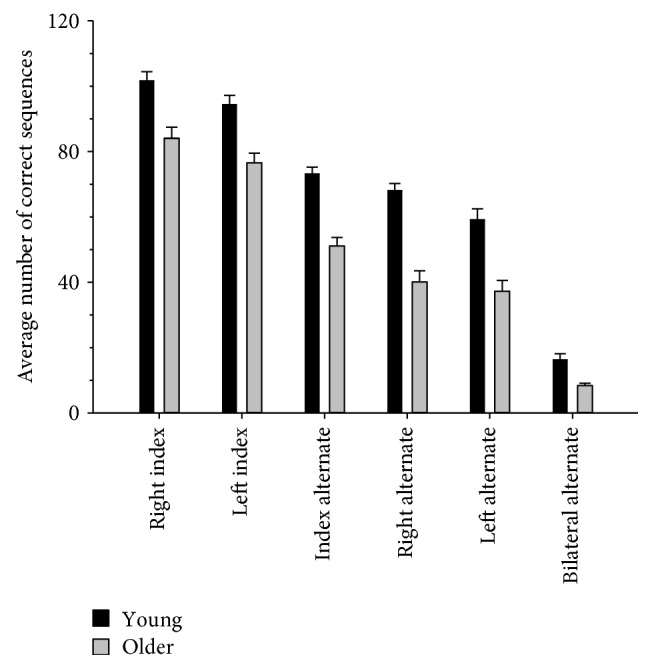
Motor performance on the six different tapping tasks in young (*n* = 18) and older (*n* = 20) adults. The number of correct motor sequences completed was significantly reduced in older compared with younger adults. Overall performance, averaged across the two age groups, declined with tasks involving greater complexity. Error bars denote SEM.

Figure 2 displays the performance of young and older adults on the six tapping tasks testing unilateral and bilateral motor functioning. Performance decreased with increasing task complexity for both young and older adults, an effect which proved reliable with a significant main effect of task (*F* (5,180) = 435.73, *p* < .001, η_p_^2^ = .92). Overall performance was poorer in older relative to young adults, as indicated by a main effect of age (*F* (1, 36) = 41.23, *p* < .001, η_p_^2^ = .53).

Performance across the tasks also varied as a function of age. This effect was found to be reliable with a significant task × age interaction (*F* (5,180) = 5.89, *p* < .001, η_p_^2^ = .14). Follow-up *t*-tests compared performance on each of the different tasks separately in young and older adults. Performance decreased significantly as the tasks became increasingly more complex. Older adults and younger adults showed the same pattern of results with a significant difference between all tasks (*p*s < .003) other than between the right alternate and left alternate tasks (old, *t* (19) = 2.03, *p* = .056, *d* = 0.45; young, *t*(17) = 2.33, *p* = .032, *d* = 0.55). However, in difference to older adults, young adults did not show a significant difference between the index alternate and the right alternate (*t* (17) = 2.53, *p* = .022, *d* = 0.60) tasks, when adjusting for multiple comparisons (adjusted alpha *p* = 0.003).

### 3.3. Spatial Attention Task Accuracy

For the spatial attention task undertaken during PAS, overall accuracy was high and few errors were made, suggesting that participants were maintaining attention effectively for the duration of the task. There was no significant difference in total errors between the attend right (M = 1.10, SE = 0.05) and the attend left conditions (M = .65, SE = 0.03), but ANOVA did reveal a weak trend (*F* (1, 38) = 3.62, *p* = .065, η_p_^2^ = .087). There was also no significant difference in the number of errors made by young (M = .95, SE = 0.05) and older adults (M = .80, SE = 0.05) and no interaction between age and attention (*p*s > .530).

### 3.4. Baseline Corticospinal Excitability

Baseline MEPs did not differ across conditions (Table 1). ANOVA did reveal a weak trend toward an attention × hemisphere × age interaction (*F* (1, 38) = 3.62, *p* = .065, η_p_^2^ = .09) driven by slightly larger baseline MEPs in the target hemisphere of young adults, but there were no other significant main effects or interactions (*p*s > .132). There were no differences in test stimulus intensities across all conditions (*p*s > .138). There was little difference in post-rMT (reported as % MSO) across the conditions, but ANOVA revealed a significant attention × hemisphere × age interaction (*F*(1, 38) = 6.89, *p* = .012, η_p_^2^ = .15). This effect was driven by slightly higher rMTs in the target hemisphere in the attend left condition in older adults. Follow-up 2 × 2 ANOVAs with the factors of attention (right and left) and hemisphere (target and nontarget) were conducted separately for young and older adults. It was found that rMTs in young adults did not differ between the conditions, as indicated by the absence of any main effects of interactions (*p*s > .179). In older adults, however, rMTs in the target hemisphere varied across the two sessions, as indicated by a significant interaction between attention and hemisphere (*F* (1, 19) = 5.33, *p* = .032, η_p_^2^ = .219). Specifically, in older adults, rMT did not differ between the attend left and attend right sessions in the nontarget hemisphere (*t* (19) = .110, *p* = .914, *d* = 0.03), but the target hemisphere rMT was significantly greater in the attend left session than in the attend right session (*t* (19) = 2.50, *p* = .022; after correcting for multiple comparisons, *d* = 0.56). This difference, however, was less than 2% MSO. Importantly, post-PAS rMTs did not differ from baseline rMTs (all other main effects and interactions, *p*s > .144).

### 3.5. PAS-Induced Corticospinal Plasticity

**Figure 3:**
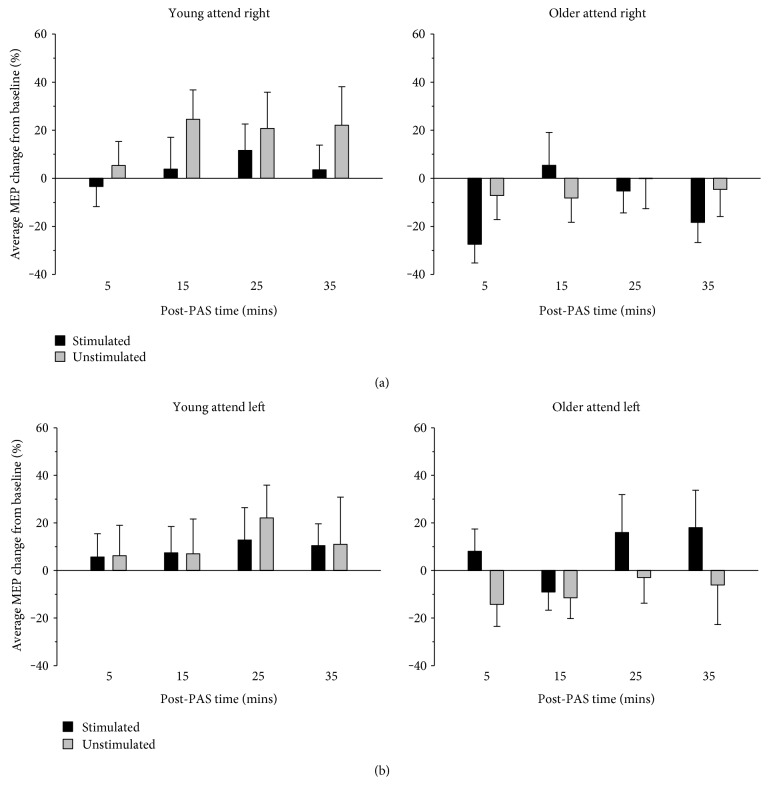
Normalized MEP changes following PAS. The amplitude of MEPs following PAS was not affected by age. The percentage of MEP change post-PAS relative to baseline did not differ significantly between the stimulated and unstimulated hemispheres nor between the attend left and attend right conditions. Error bars denote SEM.

There was no reliable difference in MEP change between young and older adults (Figure 3) with only a weak trend toward a main effect of age (*F* (1, 38) = 3.18, *p* = .083, η_p_^2^ = .077) whereby young adults tended to show increases in MEP amplitude and older adults, decreases. Moreover, follow-up analysis revealed that MEP change in each group was not significantly different from zero (*p*s > .136). There was also no reliable difference in MEP change across the post-PAS measures, only a trend toward a main effect of time (*F* (3,114) = 2.64, *p* = .053, η_p_^2^ = .065). The largest difference between the time points, which was between 5 and 25 mins post-PAS, was not statistically reliable (*t* (39) = 2.58, *p* = .014, *d* = 0.41, all other *p* > .111; adjusted alpha = .008). Furthermore, MEP change was not significantly different from baseline at any of the timepoints post-PAS (*p*s > .100). There were no other significant main effects or interactions (*p*s > .146).

Although there were no PAS-induced effects at the group level, there was substantial individual variability in response to PAS. Analysis of individual responses revealed that only half of all participants demonstrated the predicted increase in MEP amplitude averaged across all post-PAS timepoints in the target muscle; this was true for both the attend right (20 total: 12 young, 8 older; 9 females, 11 males) and attend left (22 total: 11 young, 11 older, 7 females, 15 males) sessions. The other half of the participants demonstrated a decrease in MEP amplitude. To explore these effects, participants were classified into two groups: individuals demonstrating an increase in MEP amplitude (>10% increase; LTP-like responders) and those showing a decrease (>10% decrease; LTD-like responders). Participants were classified into these two groups based on responses in the target hemisphere, allowing an examination of the resultant manifestation of corticospinal plasticity in the nontarget pathway.

**Figure 4:**
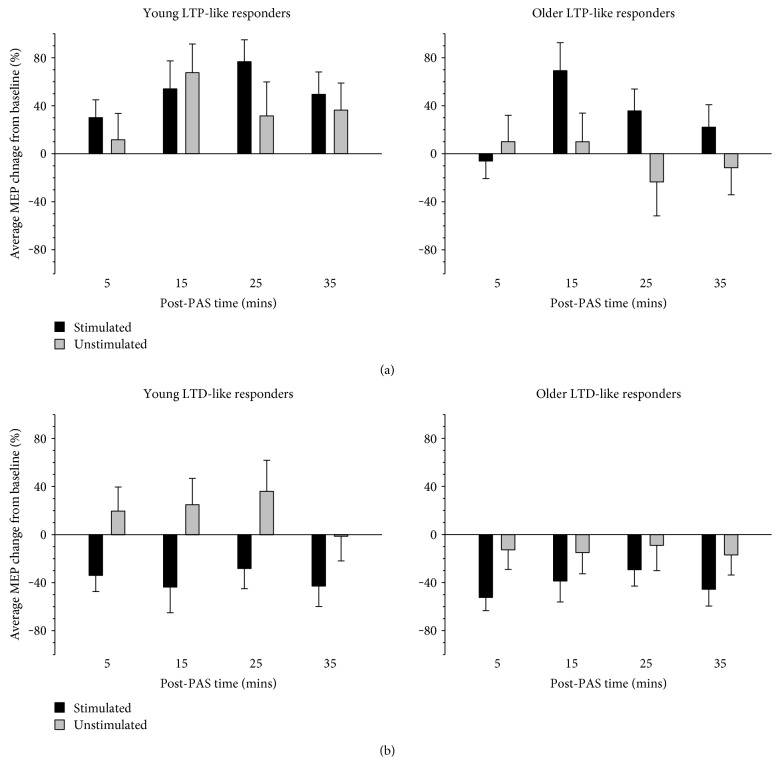
Average MEP change in young and older LTP- and LTD-like responders in the attend right condition. MEP amplitudes increased significantly in LTP-like responders and decreased significantly in LTD-like responders. Although there was no difference in the corticospinal plasticity induced in the target and nontarget motor cortices in LTP-like responders, there was a significant difference between the hemispheres in LTD-like responders. PAS-induced corticospinal plasticity was significant (different from zero) only in the target (stimulated) pathway in both LTP- and LTD-like responders. Error bars denote SEM.

As expected, in the attend right condition, MEPs increased in LTP-like responders (Figure 4(a)) but decreased in LTD-like responders (Figure 4(b)), a difference which proved reliable with a main effect of response type (*F* (1, 21) = 15.17, *p* = .001, η_p_^2^ = .419). Younger adults demonstrated an overall increase in MEP amplitude, whereas older adults demonstrated a decrease in MEP amplitude, which was supported by a main effect of age (*F* (1, 21) = 4.32, *p* = .050, η_p_^2^ = .170). MEP amplitude also differed across time, which was supported by a significant main effect of time (*F* (3, 63) = 2.87, *p* = .043, η_p_^2^ = .120). The largest difference was evident between MEP amplitude at 5 and 15 mins post-PAS; however, this difference was not significant after correcting for multiple comparisons (*p*s > .080). Although there was no significant interaction between age, hemisphere, and response type, the responses of young and older LTD-like responders appeared to vary, whereby young adults displayed opposite responses in the target and nontarget hemispheres but older adults showed similar responses across the hemispheres (Figure 4(b)). ANOVA revealed that MEP change in the LTP- and LTD-like groups did vary across the target and nontarget hemispheres, which was supported by a significant interaction between hemisphere and responder type (*F* (1, 21) = 13.19, *p* = .002, η_p_^2^ = .386). Specifically, MEP change did not differ between the hemispheres in LTP-like responders (*t* (9) = 1.58, *p* = .149, *d* = 0.50), but was significantly larger in the target hemisphere than in the nontarget hemisphere in LTD-like responders (*t* (14) = 3.70, *p* = .002, *d* = 0.95). There were no other main effects or interactions (all other main effects and interactions nonsignificant; *p*s > .099).

Although the preceding analysis illudes to differences in bilateral changes induced by LTP-like and LTD-like responders, it does not indicate whether the change in MEP amplitude was significant. In order to test this, one-sample *t*-tests compared MEP change in each of these conditions to zero. MEP change was significant from zero in the target but not the nontarget hemisphere for both LTP-like (target *t* (9) = 3.39, *p* = .008, *d* = 1.07; nontarget *t* (9) = 1.25, *p* = .243, *d* = 0.40) and LTD-like (target *t* (14) = 7.75, *p* < .001, *d* = 2.00; nontarget *t* (14) = .011, *p* = .991, *d* = 0.00) responders.

**Figure 5:**
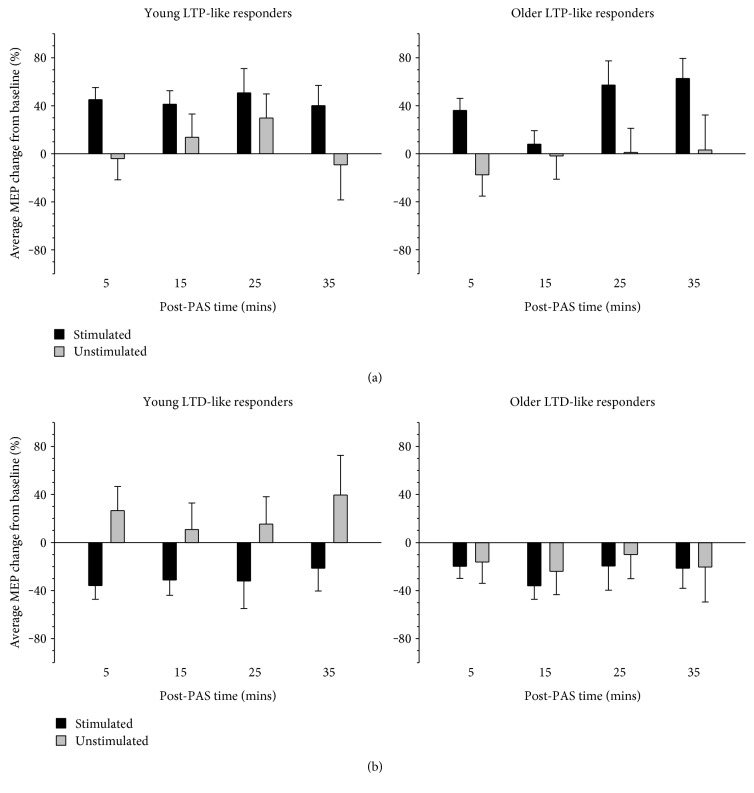
Average MEP change relative to each individual's baseline (pre-PAS MEP) for young and older LTP- and LTD-like responders in the attend left condition. MEP amplitudes increased significantly in LTP-like responders and decreased significantly in LTD-like responders. Although there was no difference in MEP amplitude change induced in the target (stimulated) and nontarget (unstimulated) hemispheres in LTD-like responders, there was a significant difference between the hemispheres in LTP-like responders. PAS-induced corticospinal plasticity was significant only in the target hemisphere in both LTP- and LTD-like responders. Error bars denote SEM.

The bilateral distribution of corticospinal plasticity effects was also examined in young and older LTP- and LTD-like responders for the attend left session (Figure 5). As expected, and similar to the effects evident in the attend right session, LTP-like responders demonstrated an increase in MEP amplitude, whereas LTD-like responders demonstrate a decrease. The effects in each responder group were reliably different from one another, as indicated by a significant main effect of response type (*F* (1, 30) = 8.27, *p* = .007, η_p_^2^ = .216). ANOVA revealed a significant interaction between hemisphere and responder type, *F*(1, 30) = 11.88, *p* = .002, η_p_^2^ = .284, but there were no other significant main effects or interactions (*p*s > .163). Follow-up analysis showed that MEP change was greater in the target hemisphere than in the nontarget hemisphere in LTP-like responders (*t* (17) = 2.90, *p* = .010, *d* = 0.68), but did not differ between the hemispheres in LTD-like responders (*t* (15) = 1.80, *p* = .093, *d* = 0.45). To assess whether the MEP change in the two hemispheres was statistically reliable, one-sample *t*-tests compared MEP change in each of these conditions to zero. MEP change was significant in the target hemisphere but not in the nontarget hemisphere for both LTP-like (target *t* (17) = 4.83, *p* < .001, *d* = 1.14; nontarget *t*(17) = .14, *p* = .887, *d* = 0.03) and LTD-like (target *t*(15) = 8.88, *p* < .001, *d* = 2.22; nontarget *t* (15) = 0.01, *p* = .989, *d* = 0.00) responders.

## 4. Discussion

The aim of this study was to determine if the manifestation of PAS-induced plasticity across bilateral motor cortices differed between young and older adults. This was assessed by comparing the effects of PAS on corticospinal excitability within the hemisphere targeted by PAS and in the nontargeted hemisphere (homologous M1) of young and older adults. We hypothesised that, compared with young adults, older adults would show greater bilateral PAS-induced corticospinal plasticity, due to age-related reductions in the lateralisation of neural activity. However, no significant PAS-induced corticospinal plasticity effect was evident at the group level, which is similar to previous reports utilizing PAS [34] and other types of noninvasive brain stimulation techniques such as iTBS [13]. This was presumably due to substantial individual variability in the PAS-induced effects. To further explore individual responses to PAS, participants were divided according to those that showed the predicted increase in corticospinal excitability following PAS (LTP-like responders) and those that showed changes in corticospinal excitability in the opposite direction (LTD-like responders). Importantly, after splitting participants into LTP-like and LTD-like responders, significant changes in corticospinal excitability were found predominately in the target hemisphere. Contrary to our hypotheses, these results suggest that PAS-induced corticospinal plasticity does not manifest bilaterally, nor is it affected by an advancing age. Regarding cognitive and motor performances, the performance of older adults was reduced compared with young adults for the Stroop, logical memory recall, and motor performance tasks, which is consistent with typical effects observed with adults around 69 years of age with an average to high level of education [35–37].

### 4.1. Age-Related Differences in PAS-Induced Effects across Bilateral Cortices

Although there was a trend toward a difference in PAS-induced effects in older and young adults, this difference was not reliable at the group level. Importantly, when looking at the LTP-and LTD-like responders separately, there was little evidence of altered corticospinal plasticity in older adults. Consistent with the findings of Dickins and colleagues [13], significant plasticity effects were limited to the targeted hemisphere, in both LTP-like and LTD-like responders, irrespective of age. This indicates that PAS-induced plasticity is not altered by advancing age and primarily manifests unilaterally in the targeted motor cortex and not in homologous regions of the nontargeted hemisphere. This is consistent with other studies using TMS to induce corticospinal plasticity in the motor cortices of young and older adults [11, 38, 39]. However, this result does not fit with the wider body of literature from human [40–42] and nonhuman animal models [43–45] demonstrating attenuated neuroplasticity in the aged brain. Similar plasticity responses to PAS were found in the present study across the two age groups, despite the older adults demonstrating clear declines in several cognitive and motor assessments. Older adults performed worse on the cognitive and motor assessments compared with their younger counterparts in the current study, suggesting this sample was experiencing some level of age-related neurobiological decline. Given that PAS responses did not differ as a function of age, this may suggest that changes in the circuits governing the cognitive, not the motor, component of voluntary motor action are contributing to declines in motor performance. However, it is also possible that the MEPs measured in this study, which reflect gross activity of both excitatory and inhibitory synapses, may not have detected more subtle changes in the cell assembly. For example, voluntary motor action is linked to the activity of gamma-aminobutyric acid (GABA; [46]). Moreover, GABA-mediated extrasynaptic inhibition is reduced in the older adult brain [47]. This suggests that the effect of advancing age might be better probed by looking at more discrete changes in cortical microcircuitry that could not be detected with the single-pulse MEP measure used in this experiment. This might be particularly informative when comparing between LTP- and LTD-like responders. Future studies would benefit from using paired pulse TMS techniques (see [48]) to better understand age-related differences in the balance of excitation and inhibition in these circuits.

### 4.2. The Role of Attention in Modulating PAS-Induced Plasticity

Another key question in the current study was whether varying the allocation of spatial attention to the target versus the nontarget hand would influence the manifestation of plasticity induced by PAS across the hemispheres in young and older adults. Previous research has reported that corticospinal plasticity can be enhanced when attention is directed to the limb targeted by PAS [15–19, 49], but no studies to date have investigated the interaction between the allocation of spatial attention and the manifestation of plasticity across the hemispheres. Importantly, although there were greater errors made in the attend right condition, accuracy was high and few errors were made overall, indicating that participants were engaged in the task. Error rates did not differ significantly between young and older adults. Attention to the target hand versus the nontarget hand did not significantly impact the magnitude of corticospinal plasticity at the group level.

Interestingly, there were no differences in the magnitude of cortical plasticity induced between the attend right and attend left sessions, suggesting that attention does not modulate the magnitude of corticospinal plasticity. This finding does not lend support to our hypothesis and is not consistent with the findings of studies investigating the modulatory role of attention in TMS-induced corticospinal plasticity [15–19, 49]. The lack of a role of attention in modifying PAS-induced plasticity is somewhat difficult to interpret, however, as there were no PAS-induced effects at the group level. Corticospinal plasticity has been shown to be modulated by attention in studies implementing visual and tactile attention manipulations [18, 19]. In contrast to Kamke and colleagues [19], the current study required participants to perform an overt visual attention task located on the thumb, wherein participants verbally responded each time they detected a target. Kamke and colleagues [19] instead required participants to perform a covert attention task wherein participants silently counted the number of targets and reported this number at the end of each trial. It is possible that the modified version used in the current experiment was too easy and did not engage sufficient attentional resources to alter the magnitude of PAS-induced corticospinal plasticity. More specifically, by using a trial-by-trial design, the previous work may have ensured that attention was directed toward the hand every time the paired stimuli of PAS occurred, which is not guaranteed in the present study. Future research might consider varying the complexity of concurrent attention manipulations to assess the degree to which the modulation of corticospinal plasticity by attention is dependent on the attention task in an attempt to identify the ideal attentional/task conditions in which LTP-like corticospinal plasticity is enhanced by attention (cf., [15]). Additionally, overtly attending to the location of the target muscle may have involved different attentional resources to that used during the covert attention task, which may also have contributed to the inconsistency in the findings. At the very least, the current results together with the wider literature highlighting attention effects [15, 18, 19] suggest that attention should be controlled in the future studies investigating PAS-induced corticospinal plasticity in young and older adults.

### 4.3. Individual Variability in PAS-Induced Corticospinal Plasticity

As noted above, there was considerable interindividual variability in the magnitude and direction of PAS-induced corticospinal plasticity, with only half of all participants demonstrating the expected increase in MEP amplitude. Variability in responses to TMS-based neuroplasticity interventions has been reported previously [31, 41, 50–53]. Several factors have been shown to contribute to this variability (see [54] for review), some of which were controlled in the current study. For example, neither age nor gender accounted for the variability in responses in the current study as the proportions were approximately equivalent across LTP-like and LTD-like responder groups. Additional analyses (not reported) demonstrated that neither physical nor sedentary activity were correlated with corticospinal plasticity, which is contrary to what has been shown previously with self-reported physical activity and TMS-induced plasticity [55]. Time of day was counterbalanced across individuals but held constant within individuals across testing sessions to eliminate variability due to diurnal fluctuations in cortisol [31]. Furthermore, active motor threshold (aMT) was replaced with rMT to eliminate variability in TMS-induced responses caused by voluntary muscle activity prior to the PAS intervention [56–59].

Although it is possible that methodological differences between the current and previous studies, such as the use of a standard interval between TMS and peripheral nerve stimulation (25 ms), or the use of a shorter PAS protocol, contributed to the variability and size of plasticity responses, evidence suggests that this is unlikely given that plasticity responses have been induced with shorter protocols and remain highly variable even when using individualized interstimulus intervals [31, 41]. Instead, factors related to the individual such as sleep, genetic polymorphisms, or nicotine consumption (see [54] for review) are more likely to have played a role. For example, slow wave sleep plays a critical role in maintaining homeostasis at a synapse (see [60] for review) and a reduction in the amount of slow wave sleep may result in insufficient normalising of synapses, which may make them less likely to undergo further potentiation in response to a neuroplasticity intervention. To reduce variability in plasticity responses, future studies would benefit from investigating the additional factors that impact responses to PAS in young and older adults.

### 4.4. LTP- and LTD-Like Responders and the Role of Attention

Although attention did not alter plasticity at the group level, when the manifestation of plasticity was assessed separately for the attend right and attend left sessions, it appeared to differ depending on the direction of MEP change observed in the target hand. When young and older participants directed spatial attention to the limb targeted by PAS (right) and demonstrated an increase in MEP amplitude in the target hand post-PAS (LTP-like responders), cortical excitability increased similarly in both hemispheres. When participants allocated attention to the target hand and demonstrated a decrease in MEP amplitude in the target hand (LTD-like responders), the decrease in cortical excitability was limited to the target hemisphere. Furthermore, when participants allocated attention to the nontarget hand (left) and demonstrated an increase in MEP amplitude in the target hand (LTP-like responder), the increase in cortical excitability was limited to the target hemisphere. But when participants allocated attention to the nontarget hand and demonstrated a decrease in MEP amplitude in the target hand (LTD-like responders), cortical excitability decreased similarly in both hemispheres. MEP change in the nontarget hemispheres, however, was not statistically reliable. Caution must be exercised when interpreting these results as the responses for the different attention conditions are compared qualitatively not quantitatively. This was because participants were classified separately for the different testing sessions, in which attention was directed to one or the other hand, due to the intersession variability in PAS-induced responses within individuals [61]. However, these findings suggest that in both young and older adults, attention directed to the target hand might facilitate bilateral LTP-like plasticity induction in people who exhibit LTP-like responses in the target hemisphere, whereas attention to the nontarget hand might facilitate bilateral LTD-like plasticity induction in people who exhibit LTD-like responses in the target hemisphere. This finding is consistent with the one other study that investigated bilateral effects induced by PAS in young adults, which found bilateral LTP-like effects at the group level that did not differ between the hemispheres [62]. The effects of overtly allocating spatial attention to the target hand versus the nontarget hand during TMS-based plasticity interventions on bilateral plasticity induction require further investigation and may have significant implications for the use of stimulation-based upper limb rehabilitation techniques with stroke survivors.

## 5. Conclusions

The current study investigated whether PAS-induced corticospinal plasticity manifests differently in the targeted and nontargeted hemispheres of young and older adults and how attention might influence this effect. There were no significant PAS-induced corticospinal plasticity effects reported in either young or older adults at the group level, but there was substantial individual variability in responses. Examining LTP- and LTD-like responders separately revealed robust PAS-induced effects occurring in opposite directions. These effects were more pronounced in the target corticospinal pathway in both young and older adults. Contrary to predictions, this result suggests that the distribution of PAS-induced corticospinal plasticity across bilateral corticospinal pathways is not altered in the aged brain. Instead, together with our previous work [12, 13], the findings indicate that aged-related change in the manifestation of plasticity is most evident when participants engage in a motor task that requires conscious voluntary movement.

The implications of this research are particularly important in light of our current ageing population. With advancing age comes increased risk of experiencing brain injury such as stroke, as well as age-related decline in motor functioning [63–65]. A greater understanding of how neuroplasticity is affected by advancing age, and how factors such as attention might modulate neuroplasticity, will have implications for the development of age-appropriate strategies to enhance motor learning in older adults. Moreover, this understanding will be important for informing motor rehabilitation, especially involving noninvasive brain stimulation, in older adults with brain injury.

## Figures and Tables

**Table 1:** Means and standard error of the means (in parentheses) of baseline corticospinal excitability and post-PAS rMTs.

	Young				Older			
	Target hem		Nontarget hem		Target hem		Nontarget hem	
	Attend right	Attend left	Attend right	Attend left	Attend right	Attend left	Attend right	Attend left
Baseline MEP (mV)	0.94	0.96	0.88	0.79	0.86	0.83	0.84	0.88
	*(0.07)*	*(0.06)*	*(0.07)*	*(0.07)*	*(0.06)*	*(0.06)*	*(0.05)*	*(0.05)*
Test intensity (% MSO)	48.15	49.15	50.15	50.05	53.30	54.60	53.75	53.55
	*(2.02)*	*(2.02)*	*(2.24)*	*(2.18)*	*(2.32)*	*(2.40)*	*(2.44)*	*(2.48)*
Baseline rMT (% MSO)	40.70	40.45	40.60	41.60	43.20	45.15	43.45	43.45
	*(1.62)*	*(1.49)*	*(1.68)*	*(1.50)*	*(1.72)*	*(1.93)*	*(1.72)*	*(1.89)*
Post-PAS rMT (% MSO)	41.00	40.55	40.75	41.30	43.05	44.75	43.75	43.60
	*(0.35)*	*(0.37)*	*(0.36)*	*(0.38)*	*(0.35)*	*(0.37)*	*(0.36)*	*(0.38)*

**Table 2:** Mean cognitive, physical, and psychological data of young and older adults. Parentheses indicate standard error of the mean.

	Young	Older	*t*-test
Stroop interference	−41.06 (3.30)	−49.95 (2.38)	*t* (36) = 2.22, *p* = .033^∗^, *d* = 0.74
Digit span test	20.61 (1.35)	20.15 (1.14)	*t* (36) = .26, *p* = .794, *d* = 0.09
Logical memory immediate recall	14.83 (0.87)	11.55 (0.79)	*t* (36) = 2.83, *p* = .008^∗^, *d* = 0.94
Logical memory delayed recall	13.28 (1.14)	10.10 (0.75)	*t* (36) = 2.37, *p* = .023^∗^, *d* = 0.79
Satisfaction with life scale	27.83 (0.94)	27.60 (0.77)	*t* (36) = .19, *p* = .848, *d* = 0.06
Multiple identities	18.94 (1.00)	15.75 (1.44)	*t* (36) = 1.79, *p* = .083, *d* = 0.60
GPAQ: average minutes per week	494.03 (66.25)	984.25 (206.73)	*t* (36) = 2.26, *p* = .034^∗^, *d* = 0.75
Sedentary behavior (mins per week)	513.33 (58.81)	378.00 (34.80)	*t* (36) = 2.16, *p* = .038^∗^, *d* = 0.72
Level of educational attainment	7.28 (0.49)	6.10 (0.55)	*t* (36) = 1.58, *p* = .123, *d* = 0.53

^∗^Significant at *p* < .05.
